# Prognostic Value of Systemic Immune-Inflammation Index in Mucosal Malignant Melanoma

**DOI:** 10.3390/jcm15020890

**Published:** 2026-01-22

**Authors:** Burak Paçacı, Erkam Kocaaslan, Ahmet Demirel, Fırat Akagündüz, Mustafa Alperen Tunç, Yeşim Ağyol, Ali Kaan Güren, Abdussamed Çelebi, Selver Işık, Ezgi Çoban, Nargiz Majidova, Nadiye Sever, Işık Paçacı, Buket Erkan Özmarasali, Adem Deligönül, Ali Fuat Gürbüz, Melek Karakurt Eryılmaz, Şüheda Ataş İpek, Nisanur Sarıyar Busery, Emre Yılmaz, Murat Sarı, İbrahim Vedat Bayoğlu, Osman Köstek, Nazım Can Demircan

**Affiliations:** 1 Division of Medical Oncology, Department of Internal Medicine, Faculty of Medicine, Marmara University, Istanbul 34854, Turkey; erkamkocaaslan@gmail.com (E.K.); drahmetdemirel23@gmail.com (A.D.); fratakagunduz0@gmail.com (F.A.); m.alperen.tunc@gmail.com (M.A.T.); yesimagyol@gmail.com (Y.A.); alikaanguren@gmail.com (A.K.G.); abdussametcelebi@gmail.com (A.Ç.); dr-selver83@hotmail.com (S.I.); ezgi.yuzugullu@gmail.com (E.Ç.); nergiz.mecidova1991@gmail.com (N.M.); dr.nadya@hotmail.com (N.S.); drmuratsari@gmail.com (M.S.); drvebay@gmail.com (İ.V.B.); 2 Department of Internal Medicine, Faculty of Medicine, Marmara University, Istanbul 34854, Turkey; isikarabakal@gmail.com; 3 Division of Medical Oncology, Department of Internal Medicine, Faculty of Medicine, Bursa Uludag University, Bursa 16059, Turkey; buketerkan@uludag.edu.tr (B.E.Ö.); ademd@uludag.edu.tr (A.D.); 4 Division of Medical Oncology, Department of Internal Medicine, Faculty of Medicine, Necmettin Erbakan University, Konya 42080, Turkey; dr.alifuatg@gmail.com (A.F.G.); drangelkarakurt@hotmail.com (M.K.E.); 5 Division of Medical Oncology, Department of Internal Medicine, Faculty of Medicine, Balcali Hospital, Cukurova University, Adana 01790, Turkey; suhedaatas92@gmail.com; 6 Division of Medical Oncology, Department of Internal Medicine, Kartal Dr. Lutfi Kirdar City Hospital, Istanbul 34865, Turkey; sariyarnisanur@gmail.com; 7 Division of Medical Oncology, Department of Internal Medicine, Faculty of Medicine, Sakarya University, Sakarya 54100, Turkey; emreyilmz@gmail.com

**Keywords:** mucosal melanoma, Systemic Immune-Inflammation Index, prognosis, biomarker, overall survival

## Abstract

**Background**: Mucosal malignant melanoma (MMM) is a rare and aggressive malignancy with a dismal prognosis. While the Systemic Immune-Inflammation Index (SII) has emerged as a prognostic marker in various solid tumors, its specific value in MMM remains undefined. This study investigated the association between pretreatment SII and overall survival (OS) in patients with MMM. **Methods**: We retrospectively analyzed 106 adults with histologically confirmed MMM treated at six oncology centers in Turkey between 2005 and 2025. The baseline SII was calculated as platelet × neutrophil/lymphocyte counts obtained before definitive treatment. A receiver operating characteristic (ROC) analysis identified an optimal SII cutoff of 776 for overall survival (OS), defining low (<776) and high (≥776) SII groups. **Results**: Gastrointestinal and head and neck mucosa were the most frequent primary sites, and one-third of patients presented with metastatic disease. The median OS for the entire cohort was 23.3 months. Patients with a high versus low SII had a shorter OS (16.2 vs. 35.2 months; HR 2.71, 95% CI 1.67–4.40; *p* < 0.001). In multivariable analysis, a high SII (HR 1.88, 95% CI 1.12–3.14; *p* = 0.016), gastrointestinal primary site (HR 1.99, 95% CI 1.23–3.23; *p* = 0.005), and metastatic disease at diagnosis (HR 4.01, 95% CI 2.32–6.94; *p* < 0.001) independently predicted a worse OS. **Conclusions**: The SII is a novel, independent prognostic biomarker in MMM. Elevated pretreatment SII correlates with aggressive clinicopathologic features and inferior survival. As a readily accessible and cost-effective marker, SII may facilitate improved risk stratification in routine clinical practice for MMM patients.

## 1. Introduction

Mucosal malignant melanoma (MMM) is a rare and biologically aggressive melanoma subtype arising from melanocytes of the aerodigestive and genitourinary mucosae, accounting for <2% of all melanomas and carrying a consistently poorer prognosis than cutaneous disease [1,2]. Beyond its epidemiologic rarity, MMM displays a distinctive biology: the overall mutational burden is lower than in cutaneous malignant melanoma (CMM); BRAF V600 and NRAS alterations are infrequent, whereas KIT alterations are relatively enriched, reflecting divergent oncogenic drivers, and ultraviolet radiation is not the dominant etiologic force except in specific mucosal sites (e.g., a subset of conjunctival tumors) [3,4]. Due to its deep anatomic location and nonspecific clinical manifestations, diagnosis is often delayed, and the majority of patients present with locally advanced or metastatic disease [5]. Clinically, survival remains short despite contemporary multimodality care, with site-related heterogeneity sinonasal primaries and the advanced TNM stage repeatedly associating with inferior outcomes in pooled analyses [1]. The prognosis of MMM patients remains dismal, with the median overall survival (OS) being reported to be below 24 months [6,7].

Immune checkpoint inhibitors (ICIs) have redefined melanoma therapy; however, the activity in MMM lags behind CMM. In a pooled analysis of clinical trials, nivolumab plus ipilimumab improved efficacy relative to monotherapy; yet, the response rates and survival in mucosal cohorts were lower than in the cutaneous counterparts [8]. Independent series focusing on mucosal primaries similarly reported modest objective response rates and short median progression-free survival (PFS) with anti-PD-1 therapy [9]. These observations underscore the substantial inter-patient variability in MMM outcomes and motivate the search for robust, accessible biomarkers to refine the risk stratification.

Classical clinicopathologic factors contribute only partially to this variability. Multivariable syntheses implicate the anatomical site and stage as dominant prognosticators in MMM, whereas the incremental prognostic value of histopathologic invasion descriptors such as lymphovascular and perineural invasion (LVI and PNI) is inconsistent across series and often context-dependent [1,10]. Accordingly, interest has shifted toward systemic inflammation–immunity indices derived from routine blood counts, which capture macro-level host tumor interactions at low cost and with excellent feasibility.

Among these biomarkers, the Systemic Immune-Inflammation Index (SII) integrates platelets and neutrophils (surrogates of protumoral inflammation and thrombocyte-driven niche support) with lymphocytes (a proxy for antitumoral immunity), calculated as platelet × neutrophil/lymphocyte [11]. Elevated neutrophil and platelet counts are associated with tumor-promoting inflammation and metastasis, whereas lymphopenia reflects impaired antitumor immunity [12,13,14]. Across solid tumors, meta-analyses have been associating elevated SII with worse OS [15]. Under ICI therapy specifically, a dedicated meta-analysis showed that a high SII portends an inferior OS and PFS in heterogeneous cancer cohorts [16]. Conversely, melanoma-focused data remain limited and mixed. In an advanced CMM cohort treated with ICIs, the baseline SII failed to retain independent prognostic significance after an adjustment for other factors, despite showing some discriminatory ability across stages [17]. Taken together, these gaps justify the tumor-type-specific evaluation.

Evidence regarding the prognostic impact of SII in mucosal malignant melanoma remains extremely limited. Given the biological heterogeneity and aggressive nature of MMM, identifying accessible and reliable prognostic markers is crucial for risk stratification and individualized management. Moreover, classical histopathologic factors such as tumor stage, LVI, PNI, and primary tumor location have been variably associated with prognosis in mucosal melanoma. While an advanced stage and certain anatomical sites (e.g., sinonasal) consistently portend inferior survival, the prognostic impact of LVI/PNI has been inconsistent across series and may be context-dependent (e.g., LVI predicting nodal metastases in lower-genital-tract melanomas) [1,10,18,19].

Since MMM is biologically and clinically distinct from CMM, extrapolating inflammatory indices across subtypes and treatment contexts risks miscalibration. In light of these gaps, we investigated whether pretreatment SII is associated with survival and provides independent prognostic information beyond established clinicopathologic variables in MMM.

## 2. Materials and Methods

### 2.1. Study Design and Population

This retrospective, multicenter cohort study included adult patients (≥18 years) diagnosed with mucosal malignant melanoma (MMM) and treated across six oncology centers in Turkey between 2005–2025. All patients had histopathologically confirmed MMM originating from mucosal sites of the head and neck, and gastrointestinal (GI) or genitourinary tracts. Patients with missing clinical data, baseline laboratory parameters, and active infection and hematological disease were excluded. The study protocol was approved by the institutional review board of Marmara University Pendik Training and Research Hospital and was conducted in accordance with the principles of the Declaration of Helsinki (ethics approval no. 09.2025.25-1018).

### 2.2. Data Collection and Variables

Demographic, clinical, and pathological data were retrospectively extracted from patient files and institutional electronic medical records. These data included age, sex, primary tumor location, tumor size, disease stage at diagnosis, presence of LVI, PNI, baseline complete blood count, and lactate dehydrogenase (LDH) obtained prior to definitive treatment. Performance status was assessed with Eastern Cooperative Oncology Group (ECOG) scale. Disease stage was stratified as local, locally advanced, and metastatic. Local disease was defined as tumor without regional lymph node or distant metastasis. Locally advanced disease was defined as regional lymph node metastasis without distant metastasis. Metastatic stage corresponded to distant metastatic disease. OS was defined as the interval in months between the date of diagnosis and the date of death from any cause or last follow-up.

Molecular testing results (BRAF, KIT, and NRAS) were obtained from official pathology and molecular diagnostics reports archived in the institutional electronic medical records. Testing was performed in accredited molecular pathology laboratories at the participating centers or external reference laboratories, using clinically validated assays (e.g., real-time PCR, and next-generation sequencing panels) depending on the calendar year and local availability.

SII was calculated as follow:

“SII” = (“Platelet count” × “Neutrophil count”)/“Lymphocyte count”

All counts were derived from peripheral blood samples collected at diagnosis and prior to initiation of any definitive treatment (surgery, radiotherapy, or systemic therapy); if multiple samples were available, the one closest to diagnosis was used. To determine the optimal cutoff value of SII predicting OS, a receiver operating characteristic (ROC) curve analysis was performed. The Youden index was applied to identify the threshold providing the highest combined sensitivity and specificity, which yielded a cutoff of 776. Patients were subsequently stratified into two groups: Low SII (<776) and high SII (≥776).

### 2.3. Statistical Analysis

Descriptive statistics were presented as median with interquartile range or frequency with group percentage. The association between categorical variables was analyzed using Chi-square test or Fisher’s exact test where applicable.

Survival was estimated using Kaplan–Meier method, and differences between groups were assessed with log-rank test. Variables significantly associated with OS in univariate analysis (*p* < 0.05), as well as those showing a trend toward significance (*p* < 0.10), were entered into a multivariate Cox regression model to identify independent prognostic factors. Hazard ratios (HRs) and 95% confidence intervals (CIs) were calculated for each covariate. A two-tailed *p*-value of <0.05 was considered statistically significant throughout all analyses. Statistical analyses were performed using IBM SPSS Statistics Version 27 (IBM Corp., Armonk, NY, USA). The proportional hazards assumption was assessed using log-minus-log survival plots.

Sensitivity analyses included (i) a Cox model stratified by treatment center to account for potential inter-institutional heterogeneity; (ii) additional models coding key pathological variables with missingness (LVI, PNI, and tumor ulceration) as separate categories (absent/present/unknown) to assess robustness; and (iii) an additional model adjusting for diagnosis era (2015–2025 vs. ≤2014) to account for temporal changes in treatment strategies.

## 3. Results

### 3.1. Patient and Tumor Characteristics

A total of 106 patients with mucosal malignant melanoma (MMM) were included in the analysis. The median age at diagnosis was 66 years (interquartile range [IQR], 57–75). The baseline demographic and clinical features according to SII groups are presented in Table 1. The cohort was evenly distributed by age, with 52.8% of patients aged ≥ 65 years. No significant associations were observed between SII level and age (*p* = 0.275), sex (*p* = 0.798), or comorbidity status (*p* = 0.668). Most patients (87.7%) had an ECOG performance status of 0 or 1. High SII values (≥776) were significantly associated with a poorer performance status (ECOG ≥ 2) (*p* = 0.037). Regarding the primary tumor site, the gastrointestinal mucosa represented the most common location (45.3%), followed by the head and neck (40.6%). A trend toward higher SII levels was observed in gastrointestinal primaries compared with non-gastrointestinal sites, although this did not reach statistical significance (*p* = 0.062). At diagnosis, 41.5% of patients had local, 25.5% had locally advanced, and 33% had metastatic disease. A high SII was significantly correlated with an advanced stage at presentation (*p* = 0.003). The presence of LVI was also associated with an elevated SII (*p* < 0.001), whereas PNI and tumor ulceration were not significantly related to SII status (*p* = 0.115 and *p* = 0.122, respectively). Among all patients, 60 (56.6%) underwent surgical resection (R0 = 49, and R1 = 11), while 46 (43.4%) either had an R2 resection or were not operated on. High SII values were significantly associated with an incomplete or absent resection (*p* = 0.035). The metastatic involvement of specific organs (lung, liver, bone, brain, or other sites) did not differ significantly between SII groups (*p* > 0.05 for all). The treatment patterns were heterogeneous: in the adjuvant setting, chemotherapy alone was the most common modality (*n* = 21), while concurrent chemoradiotherapy alone was administered to 8 patients, 2 patients received adjuvant immune checkpoint inhibitors, and none received targeted therapy. In the metastatic setting, first-line systemic therapy consisted of chemotherapy (*n* = 54), immune checkpoint inhibitors (*n* = 20), and targeted therapy (*n* = 1).

### 3.2. Mutational Analysis

The genetic analyses are summarized in Table 2. Among 84 patients with available molecular data, BRAF mutations were identified in 11.9% (10/84) and showed a significant association with high SII levels (*p* = 0.019). In contrast, c-KIT and NRAS mutations demonstrated no statistically significant correlation with the SII group (*p* = 0.678 and *p* = 1.000, respectively).

### 3.3. Survival Analysis

Among all 106 patients, 72 deaths occurred during follow-up. The median overall survival (OS) was 23.3 months, and the 5-year OS rate was 23%.

### 3.4. Univariate Survival Analysis

**Figure 1:**
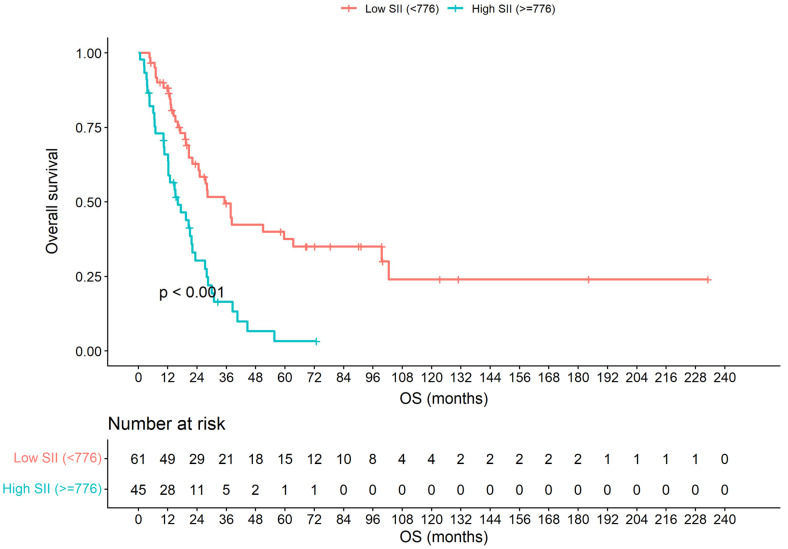
Kaplan–Meier estimates of overall survival according to the Systemic Immune-Inflammation Index (SII) (high SII group: ≥776, low SII group: <776; log-rank test *p* < 0.001). Numbers at risk are shown below the plot.

The univariate Cox regression results for OS are detailed in Table 3. None among the age (*p* = 0.471), sex (*p* = 0.247), comorbidity (*p* = 0.935) and baseline LDH level (*p* = 0.913) showed prognostic significance. Conversely, the disease stage, mucosal site, LVI status, and SII group were all significantly associated with OS. Patients presenting with metastatic disease exhibited a markedly shorter OS compared with those with non-metastatic disease (10.3 vs. 28.4 months; HR 5.13, 95% CI 3.08–8.56; *p* < 0.001). Gastrointestinal primaries were associated with inferior survival compared with non-gastrointestinal sites (16.1 vs. 30.1 months; HR 2.28, 95% CI 1.42–3.64; *p* < 0.001). Similarly, the presence or uncertainty of LVI predicted poorer outcomes (HR 2.03, 95% CI 1.13–3.66; *p* = 0.015). Importantly, patients with an elevated SII (≥776) had a significantly shorter OS (median 16.2 months) than those with a low SII (<776; median 35.2 months; HR 2.71, 95% CI 1.67–4.40; *p* < 0.001), with corresponding 5-year OS rates of 3% and 38%, respectively (Figure 1).

### 3.5. Multivariate Survival Analysis

The variables with *p* < 0.10 in the univariate analysis were included in the multivariate Cox regression model (Table 3). After adjustment, three variables remained independently associated with OS:

- High SII (≥776) (HR 1.88, 95% CI 1.12–3.14; *p* = 0.016);
- Gastrointestinal primary site (HR 1.99, 95% CI 1.23–3.23; *p* = 0.005);
- Metastatic disease at diagnosis (HR 4.01, 95% CI 2.32–6.94; *p* < 0.001).

Thus, a high SII index independently predicted a worse OS, irrespective of the primary tumor site and stage.

The sensitivity analyses supported the robustness of the primary findings. In a multivariable Cox model stratified by the treatment center (six strata), a high SII remained independently associated with a shorter overall survival (HR 2.30, 95% CI 1.25–4.23; *p* = 0.008), consistent in direction with the primary model. When key pathological variables were remodeled as three-level categories to account for missingness (absent/present/unknown), none showed an independent association with overall survival (LVI: overall *p* = 0.988; PNI: overall *p* = 0.917; tumor ulceration: overall *p* = 0.861), and the association between a high SII and worse survival remained materially unchanged (LVI-adjusted: HR 1.88, 95% CI 1.12–3.14; *p* = 0.016; PNI-adjusted: HR 1.87, 95% CI 1.10–3.18; *p* = 0.020; ulceration-adjusted: HR 1.92, 95% CI 1.14–3.24; *p* = 0.014). Finally, an adjustment for the diagnosis era (2015–2025 vs. ≤2014) did not attenuate the prognostic effect of a high SII (HR 1.91, 95% CI 1.14–3.19; *p* = 0.013), while the later era showed a borderline protective association (HR 0.59, 95% CI 0.34–1.00; *p* = 0.050).

## 4. Discussion

MMM is a rare and distinct subtype of melanoma, accounting for just over 1% of all melanoma diagnoses. In stark contrast to its cutaneous counterpart, MMM is characterized by a significantly more aggressive clinical course and a poorer prognosis, often attributed to its occult primary location, which frequently leads to diagnosis at an advanced stage. The clinical management of MMM remains challenging due to the lack of standardized therapeutic protocols and a notable scarcity of robust, validated prognostic biomarkers [20].

Systemic inflammation is a known hallmark of cancer that influences prognosis [21,22]. In this multi-center retrospective study, we identified the SII as a novel, independent prognostic factor for OS, alongside the metastatic disease stage and a GI primary site. To the best of our knowledge, this is the first study to specifically demonstrate the independent prognostic value of SII in a comprehensive cohort of patients with mucosal melanoma.

The relationship between inflammation and cancer progression is critical, as it influences tumor proliferation, angiogenesis, and the cancer’s evasion of the immune system [21,22]. This relationship appears particularly relevant in MMM, where inflammatory markers may predict prognosis [23]. The genomic landscape of MMM further distinguishes it from CMM. MMM typically has a lower overall mutational burden. Compared to CMM, it possesses a distinct genomic profile, characterized by a lower frequency of BRAF mutations—consistent with our finding of 11.9%—and a lower frequency of NRAS mutations, but a significantly higher prevalence of oncogenic drivers in c-KIT [4]. While the prognostic role of inflammation is well-documented in CMM and other solid tumors, the data for MMM is limited. Prior studies have validated the utility of specific inflammatory scores in CMM, including the SII and the Neutrophil-to-Lymphocyte Ratio (NLR) and Platelet-to-Lymphocyte Ratio (PLR) [24,25]. Furthermore, prior studies have validated the utility of inflammatory scores in other malignancies that arise from the head and neck and gastrointestinal tract, which represent the most common primary sites for MMM [26,27]. Our work extends these findings by focusing on the SII, a more comprehensive composite marker. We established our SII cutoff value of 776 via ROC analysis. This value is consistent with the wide range of cutoffs reported in the literature for other malignancies, which vary broadly based on the cancer type and patient population, as highlighted in several studies [26,28]. The strength of the SII lies in its utility as a simple, universally available, and cost-effective biomarker derived from a routine complete blood count, which can be readily integrated into clinical practice for risk stratification.

In exploratory analyses of the subset with available molecular testing, BRAF mutation status was associated with a higher SII, whereas KIT and NRAS alterations were not. Given the limited number of molecularly profiled patients and the small size of individual mutation subgroups, these findings should be considered hypothesis-generating and may reflect sampling variability and/or confounding by site, disease burden, or treatment era. Future studies with broader, standardized molecular profiling are warranted to clarify whether specific oncogenic drivers in MMM are linked to distinct systemic inflammatory phenotypes.

We emphasize that the optimal SII cutoff in the present study (776) was derived within our cohort and should be interpreted as cohort-specific rather than universally applicable. The reported SII thresholds vary substantially across tumor types and treatment contexts; in melanoma and immunotherapy-treated cohorts, cutoffs are frequently defined empirically (e.g., median-based) or via ROC analyses. For instance, in an ICI-treated melanoma cohort, the median SII (924) was used as a cutoff and a high SII was independently associated with inferior survival [29]. In an ipilimumab-plus-nivolumab-treated cohort, an ROC-derived SII cutoff of 788 was reported, closely aligning with the range observed in our study [30].

In our analysis of baseline characteristics, patients with a high SII (≥776) presented with a significantly worse ECOG performance status, a higher incidence of metastatic disease at diagnosis, and a higher frequency of LVI, and were less likely to achieve an R0 resection (all *p* < 0.05). These findings are not unexpected; they reflect a logical association between a heightened systemic inflammatory state and a more aggressive tumor biology. A high SII, indicating elevated neutrophils and platelets relative to lymphocytes, suggests a microenvironment that is simultaneously pro-tumorigenic and immunosuppressed. This systemic state is biologically linked to adverse pathological features. For instance, the association between a high SII and the presence of LVI has been documented in other tumor types, such as gastroesophageal adenocarcinoma, where systemic inflammation is thought to promote processes facilitating vascular invasion [28]. Similarly, our data revealed a strong link between a high SII and a lower rate of R0 resection. This finding is biologically plausible, as a high preoperative SII has been widely established as a reliable marker for a more advanced T-stage in various solid tumors [31]. A more advanced T-stage is naturally associated with a lower likelihood of achieving a complete surgical (R0) resection.

This study is limited by its retrospective design, which may introduce selection bias. Several pathological variables (LVI, PNI, and tumor ulceration) were missing in a substantial proportion of cases due to heterogeneous and historically incomplete reporting; although modelling “unknown” as a separate category in sensitivity analyses did not materially change the association between a high SII and worse survival, residual confounding related to unmeasured clinicopathological factors cannot be fully excluded. Molecular testing for BRAF, c-KIT, and NRAS was not available for the entire cohort, limiting the power for mutational subgroup comparisons. Finally, SII is a non-specific hemogram-derived marker; despite the exclusion of active infections and hematologic disease, subclinical inflammatory states or comorbidities may still influence SII and should be considered when interpreting its prognostic value. The multicenter design and long inclusion period (2005–2025) may introduce inter-institutional and temporal heterogeneity in treatment strategies, pathological assessment, and follow-up practices. To address these concerns, we performed center-stratified and era-adjusted sensitivity analyses, which supported the robustness of our primary findings. Nevertheless, external validation in independent MMM cohorts remains warranted, ideally within more uniform treatment eras and with standardized pathological and molecular reporting.

## 5. Conclusions

In conclusion, our study identifies the SII as a novel, independent predictor of OS in patients with mucosal melanoma. We propose that the SII can be integrated into routine clinical practice to enhance prognostic stratification and aid in risk assessment for this aggressive disease. Further prospective studies are warranted in order to validate these findings and to explore the predictive role of SII in response to systemic treatments, particularly immunotherapy, which could solidify its position as a crucial biomarker in the management of mucosal melanoma.

## Figures and Tables

**Table 1:** Baseline clinicopathologic characteristics stratified by SII group.

Variable	Cases	SII < 776	SII ≥ 776	*p* Value
Age, years, *n* (%)				0.275
<65	50 (47.2)	26 (42.6)	24 (53.3)	
≥65	56 (52.8)	35 (57.4)	21 (46.7)	
ECOG-PS, *n* (%)				0.037
0–1	93 (87.7)	57 (93.4)	36 (80)	
≥2	13 (12.3)	4 (6.6)	9 (20)	
Gender, *n* (%)				0.798
Female	51 (48.1)	30 (49.2)	21 (46.7)	
Male	55 (51.9)	31 (50.8)	24 (53.3)	
Comorbidity, *n* (%)				0.668
Absent	33 (33.1)	20 (32.8)	13 (28.9)	
Present	73 (68.9)	41 (67.2)	32 (71.1)	
Mucosal site, *n* (%)				0.062 *
Gastrointestinal	48 (45.3)	24 (39.3)	24 (53.3)	
Head and neck	43 (40.6)	29 (47.5)	14 (31.1)	
Gynecological	6 (5.7)	3 (4.9)	3 (6.7)	
Conjunctival	6 (5.7)	5 (8.2)	1 (2.2)	
Other	3 (2.8)	0	3 (6.7)	
Initial stage, *n* (%)				0.003
Local	44 (41.5)	30 (49.2)	14 (31.1)	
Locally advanced	27 (25.5)	19 (31.1)	8 (17.8)	
Metastatic	35 (33)	12 (19.7)	23 (51.1)	
LVI, *n* (%)				<0.001
Negative	27 (25.5)	24 (39.3)	3 (6.7)	
Positive	21 (19.8)	10 (16.4)	11 (24.4)	
Unknown	58 (54.7)	27 (44.3)	31 (68.9)	
PNI, *n* (%)				0.115
Negative	29 (27.4)	21 (34.4)	8 (17.8)	
Positive	11 (10.4)	7 (11.5)	4 (8.9)	
Unknown	66 (62.3)	33 (54.1)	33 (73.3)	
Tumor ulceration, *n* (%)				0.122
Negative	12 (11.3)	10 (16.4)	2 (4.4)	
Positive	32 (30.2)	19 (31.1)	13 (28.9)	
Unknown	62 (58.5)	32 (52.5)	30 (66.7)	
Surgery status, *n* (%)				0.035
R0 resection	49 (46.2)	34 (55.7)	15 (33.3)	
R1 resection	11 (10.4)	7 (11.5)	4 (8.9)	
R2 or not resected	46 (43.4)	20 (32.8)	26 (57.8)	
Metastatic sites, *n* (%)				
Lung	35 (49.3)	17 (34)	18 (30)	0.727
Liver	25 (35.2)	10 (20)	15 (25)	0.420
Bone	21 (29.5)	8 (16)	13 (21.6)	0.359
Brain	7 (9.8)	3 (6)	4 (6.6)	1.000 *
Other	22 (30.9)	12 (24)	10 (16.7)	0.361

* Exact *p* value (Fisher–Freeman–Halton test, Monte Carlo). ECOG-PS, Eastern Cooperative Oncology Group Performance Status; LVI, lymphovascular invasion; PNI, perineural invasion.

**Table 2:** Analyzed mutational status.

Total Number of Analyzed Patients	Cases	SII < 776	SII ≥ 776	*p* Value
BRAF (*n* = 84), *n* (%)				
Absent	74 (88.1)	45 (60.8)	29 (39.2)	0.019 *
Present	10 (11.9)	2 (20)	8 (80)	
c-KIT (*n* = 24), *n* (%)				
Absent	15 (62.5)	10 (66.7)	5 (33.3)	0.678 *
Present	9 (37.5)	5 (55.6)	4 (44.4)	
NRAS (*n* = 10), *n* (%)				
Absent	9 (90)	7 (77.8)	2 (22.2)	1.000 *
Present	1 (10)	1 (100)	0	

* Exact *p* value (Fisher–Freeman–Halton test, Monte Carlo).

**Table 3:** Cox regression analysis of factors predicting overall survival.

		Univariate Analysis		Multivariate Analysis	
Variable	OS (Months)	HR (%95 CI)	*p* Value	HR (%95 CI)	*p* Value
Age, years					
<65	23.2	1	0.471		
≥65	21.7	1.18 (0.74–1.89)			
Gender					
Female	27.9	1	0.247		
Male	20.5	1.31 (0.82–2.09)			
Initial stage					
Non-metastatic	28.4	1	<0.001	4.01 (2.32–6.94)	<0.001
Metastatic	10.3	5.13 (3.08–8.56)			
Mucosal site					
Non-gastrointestinal	30.1	1	<0.001	1.99 (1.23–3.23)	0.005
Gastrointestinal	16.1	2.28 (1.42–3.64)			
Comorbidity					
Present	23.2	1	0.935		
Absent	22.1	1.02 (0.61–1.68)			
LVI					
Absent	37.8	1	0.015	1.08 (0.50–2.35)	0.827
Present/Unknown *	20.7	2.03 (1.13–3.66)			
PNI					
Absent	27.9	1	0.098	1.10 (0.60–2.01)	0.754
Present/Unknown *	20.7	1.56 (0.91–2.67)			
LDH					
Normal	22.1	1	0.913		
Upper limit of normal	23.2	1.02 (0.64–1.64)			
SII					
<776	35.2	1	<0.001	1.88 (1.12–3.14)	0.016
≥776	16.2	2.71 (1.67–4.40)			

LVI, lymphovascular invasion; PNI, perineural invasion; LDH, lactate dehydrogenase; SII, systemic immune-inflammation. * In the primary Cox models, LVI and PNI were coded as absent vs. present/unknown due to missingness in historical pathology reports; sensitivity analyses treating missingness as a separate category (absent/present/unknown) yielded materially unchanged results.

## Data Availability

The data that support the findings of this study are not publicly available due to privacy reasons but are available from the corresponding authors.
